# Long-Term Non-Users of Transcutaneous Auditory Implants: Thirty Years of Experience at a Single Institution

**DOI:** 10.3390/ijerph20136201

**Published:** 2023-06-22

**Authors:** Miryam Calvino, Isabel Sánchez-Cuadrado, Javier Gavilán, Luis Lassaletta

**Affiliations:** 1 Department of Otolaryngology, Hospital Universitario La Paz, IdiPAZ Research Institute, 28046 Madrid, Spain; 2 Biomedical Research Networking Centre on Rare Diseases (CIBERER), Institute of Health Carlos III, CIBERER-U761, 28029 Madrid, Spain

**Keywords:** auditory implant, non-users, cochlear implant, vibrant soundbridge, bonebridge, well-being

## Abstract

*Background:* Although it is a recognized phenomenon, there is little published in the literature on the discontinuation of auditory implant use. *Aim:* To evaluate the incidence of device non-use of transcutaneous auditory implants. *Patients and Methods*: This is a retrospective study of all living individuals (children and adults) implanted at the La Paz Hospital (Madrid, Spain) between 1992–2015, with a follow-up examination endpoint of December 2022. 356 device recipients were included: 316 with cochlear implants (CI), 22 with middle-ear implants (Vibrant Soundbridge, VSB), and 18 with bone conduction implants (Bonebridge, BB). *Results:* Nine CI recipients (2.8%) were identified as non-users (mean follow-up 15.1 ± 5.4 years). The reasons for non-use were implant failure and reimplantation rejection, lack of benefit, non-attendance of rehabilitation sessions, loss of the audio processor, and cognitive and linguistic difficulties. None of them experienced any surgical complications. Six VSB recipients (27.3%) were device non-users (mean follow-up 11.4 ± 2.1 years). All of them experienced device failure or surgical complications. To date, none of the BB recipients is a non-user (mean follow-up 8.6 ± 1.1 years). *Conclusion:* The rates of non-use of transcutaneous auditory implants vary widely between different types of implants. Given the small proportion of non-users, information on what are the predictive factors could not be determined. The reasons for non-use should be carefully documented and used to guide careful patient selection to reduce the risk of non-use in future candidates.

## 1. Introduction

The field of auditory implants has experienced substantial growth within a relatively short period of time. The first cochlear implantation (CI) in Spain was performed in 1985. By 2016, more than one thousand individuals had received a CI in this country [1]. More recent devices are the middle ear implants (MEI) such as the Vibrant Soundbridge (VSB), which was first introduced in 1996 [2], and bone conduction implants (BCI) such as the Bonebridge (BB), which followed in 2011 [3]. Over the years, the number of auditory implant recipients has steadily increased due to safer surgical procedures, and the expansion of indications, particularly for pediatric candidates, those with residual hearing or single-sided deafness, and for bilateral and bimodal implantation, has also contributed to the growing population of implant recipients [4].

Despite the well-established benefits of hearing implant use, some recipients become non-users [5,6]. Each case of implant discontinuation also represents a substantial investment of time, resources, and effort that does not lead to the anticipated outcomes. The cost of elective non-use of implants has to be accounted for in healthcare budgeting [7]. The implications of non-use are therefore significant for the recipient, for their care providers, and for society at large. For this reason, identifying the causes of implant non-use is a pressing matter. It is important to report and monitor cases of non-use, to document the causes of non-use, and to identify possible predictive factors.

In this study, we evaluated incidences of device non-usage of recipients of transcutaneous auditory implants who were implanted in a single institution within the last 30 years. The reasons for non-use, when identified, are presented. Moreover, if possible, predictors of non-use were analyzed.

## 2. Materials and Methods

### 2.1. Subjects

This retrospective study used data from all transcutaneous auditory implantations (CI, MEIs, and BCIs) in children and adults at the La Paz University Hospital (Madrid, Spain) between 1992 and 2015. The follow-up examination endpoint was December 2022.

**Figure 1:**
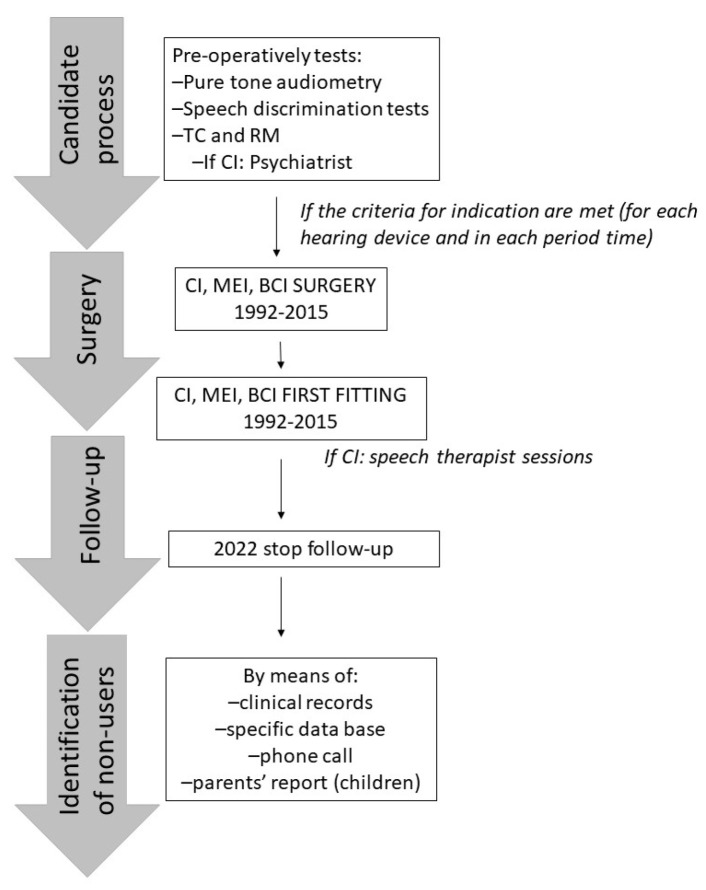
Flowchart of the process of non-users identification.

Prior to surgery, all candidates are given a thorough audiological workup. Pure tone audiometry (or auditory brainstem response in children), and speech discrimination tests in silence are performed. Radiological examinations including temporal bone computerized tomography and magnetic resonance imaging are routinely performed. If these tests indicate that an individual is a suitable candidate for CI surgery, the individual is referred to the psychiatry department to assess motivation and expectations. The decision of whether to proceed with implantation is taken after counsel with a multidisciplinary team, taking into account the indications of implantation for each case which have varied over time. Surgery is conducted by an experienced surgical team. The implants are activated within the first month post-surgery. In the case of cochlear implantation, the recipient then begins rehabilitation with speech language professionals (Figure 1).

Devices from the manufacturers MED-EL (Innsbruck, Austria), and Cochlear (Sydney, Australia) were included. If a subject was wearing a percutaneous hearing implant (Ineraid and BAHA), they were excluded from this study; this being the only exclusion criterion for the study.

### 2.2. Definition of Device Non-User

In this study, we have defined a device non-user as a recipient who has rejected the same implant usage completely. Individuals who underwent explantation and refused reimplantation were also defined as non-users, as well as subjects who lost their audio processor and could not replace it because of its high cost. Individuals who use their device but for less than the recommended number of hours per day (limited users) were not considered as non-users. This information was obtained either from clinical records or from a specific database of implant recipients; in some cases, a phone call was needed to confirm the use (or non-use) of the audio processor. In the case of children, data from parents’ reports were also necessary.

### 2.3. Variables

The following factors were specifically assessed in this study: (i) etiology of deafness; (ii) age at surgery; (iii) duration of deafness; (iv) date of surgery; (v) minor or major complications after implantation; and (vi) reasons for non-use.

Demographic details are shown as absolute (n) and relative (%) frequencies and, if appropriate, as mean plus standard deviation (±SD) and range. All data were exported into an Excel spreadsheet (Microsoft, Redmond, WA, USA), checked for accuracy and analyzed in Excel for descriptive analysis.

## 3. Results

### 3.1. Subjects

Cochlear implantation was performed in 357 patients between May 1992 and December 2015. Bilateral implantation was performed in 57 pediatric and four adult cases, therefore 418 CIs were implanted in total during this period. Of these 357 subjects, 41 (11.5%) were lost to follow-up. The reasons for loss were death (n = 26), did not attend follow-up appointments (n = 11), or their devices were explanted without reimplantation (n = 4). The remaining 316 recipients were thus considered study participants.

The MEI VSB was implanted in 22 subjects between May 2008 and December 2015.

The BCI BB was implanted in 18 subjects between June 2012 and December 2015. The demographic details of these patients are shown in Table 1.

### 3.2. CI Non-Users

Of the 316 CI recipients, 9 (2.8%) were identified as non-users. All nine non-users were adults with postlingual deafness. Their mean age at implantation was 47.2 ± 14.3 years (range 26–70 years). The mean duration of deafness was 22.3 ± 22.6 years (range 0–54 years). Their etiologies were meningitis (n = 3), unknown (n = 3), sudden hearing loss (n = 2), and otosclerosis (n = 1).

The electrode insertion was complete in all but one non-user. The types of CI that were discontinued were: Nucleus 22M (1), Combi40+ (3), Pulsar (4), and Sonata (1). Only one individual had experienced device failure (Table 2). No medical or surgical complications were observed in this case.

**Figure 2:**
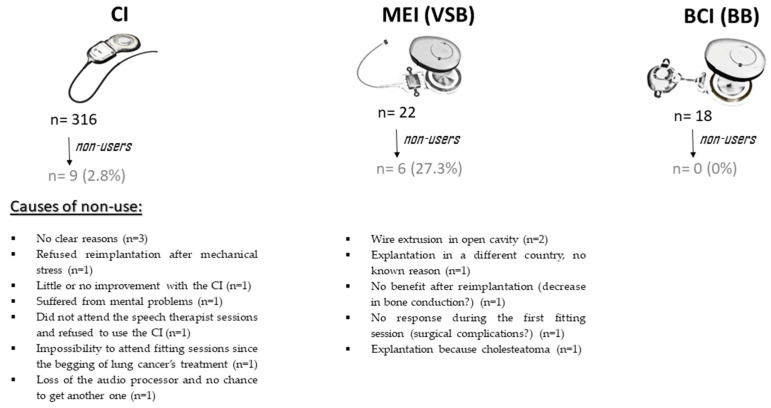
Summary of the causes of non-use of the implants. CI: cochlear implant; MEI: middle ear implant; VSB: Vibrant Soundbridge; BCI: bone conduction implant; BB: Bonebridge. Images modified from MED-EL.

The reported reasons for non-use of the implant were varied (Figure 2):

▪ Three recipients gave no clear reasons for the non-use (#1, #2, #5);
▪ One recipient refused reimplantation after mechanical stress, detected by telemetry of electrode impedances, occurred in the implant (#6);
▪ One recipient experienced little or no improvement with the CI (probably due to affected auditory nerve) (#3);
▪ One recipient suffered from mental problems (#7);
▪ One recipient with poor Spanish language skills did not attend the speech therapist sessions and refused to use the CI. This patient later moved to another country (#8);
▪ One recipient discontinued use because they began treatment for lung cancer and it became impossible (according to the recipient) to attend the fitting sessions (#9);
▪ One recipient informed us that they had lost their audio processor after 19 years of use (#4).

### 3.3. Middle Ear Implant (VSB) Non-Users

22 people received a MEI (VSB). Of these, six (27.3%) became non-users (Table 3). Non-users had a follow-up of 11.4 ± 2.1 years (range 7.2–14.6 years). All were adults. The mean age at implantation was 58.0 ± 18.0 years (range 38–77 years) vs. 49.1 ± 14.1 years (range 24–70 years) of those who were still users. All subjects had conductive or mixed hearing loss due to chronic otitis media or cholesteatoma.

In all six cases wire extrusion, lack of coupling or surgical complication occurred (Figure 2). In detail:

▪ Two recipients with implantation in open cavity experienced wire extrusion; one of them is now using a CI, and the other refused a new surgery;
▪ One recipient was explanted in a different country. The reason for this was not communicated to us;
▪ One recipient had a device coupling issue, and reimplantation was performed. However, they did not derive benefit from the reimplanted device. This was likely due to a decrease in bone conduction;
▪ One recipient did not exhibit any response during the first fitting session. This was probably due to surgical complications;
▪ One recipient had developed a cholesteatoma, requiring explantation of the VSB. This was replaced by a BB.

### 3.4. Bone Conduction Implant (BB) Non-Users

At the time of analysis, there were no BB non-users (mean follow-up 8.6 ± 1.1 years, range 10.5–7.1 years) (Figure 2).

## 4. Discussion

Implant non-use can broadly be divided into three types. Limited use is where the implant is used for less than the recommended number of hours per day [8,9]. Discontinuation is where the user has ceased to use the implant for an extended period. Permanent discontinuation is where the user has ceased using the device and affirmed that they no longer intend to use the device going forward.

In this study, we have considered only users who have completely ceased their implant use, as these represent the most serious cases, and provide the most salient information on the reasons for use cessation. An additional reason for studying only discontinuation is that older generations of audio processors, which many of our patients still wear, do not have the capability to log device usage statistics. To study cases of limited use, we would therefore have to rely upon user recall, which would likely compromise the quality of our data and the conclusions drawn from it.

To further bolster the quality of our data, we have also considered only device non-users who were not lost to follow-up. It is possible that a user can be lost to follow-up but continue using their device, perhaps under the supervision of other care providers.

Adequate monitoring of implant usage rates and counselling in the case of observed non-use should be regarded as an important part of hearing implant programs. This can help to minimize the transition from limited use to permanent non-use [6,10,11]. Moreover, in the case of children, non-use could affect the emotional investment of the family [12].

### 4.1. CI Non-Users

During the study period, the rate of CI non-users was 2.8%, all of whom were adults. In other words, 97.2% of those who were implanted and not lost to follow-up remain users of their CI. Previous studies from our group have demonstrated that these users derive substantial benefit from their devices in terms of both audiological and quality of life outcomes [13,14,15,16].

As outlined in the material and methods section, the patients included in the study were those implanted since 1992 (when the first transcutaneous CI was implanted in our center) up to the end of 2015. We have not reported results from patients who were implanted with the early generation Ineraid CI, as most of the implantees have since passed away, have been reimplanted, or we could not contact them.

In Table 4 we presented our findings in the context of previous studies which have evaluated rates of CI non-use. The median rate of implant non-use across these studies was 3.65% (range: 0–29%). The CI non-use rate reported here (2.8%) is within this range. In this study we had a follow-up period of 30 years. This is the longest follow-up period yet reported in a study on CI non-use. Of the previous studies for which reporting periods were documented, the median follow was 8 years (range 2–15 years). As such, our data may be representative of the expected rates of CI non-use in the long term. At 357 users, our study is also one of the largest cohorts yet studied on this topic; previous studies ranged from 27 to 423 subjects.

Six of the previous studies were carried out with pediatric users. Four studies were carried out with adult users. Only two were carried out in mixed cohorts of both children and adult users (as in the present study). Higher levels of non-use have tended to be observed in children. In a study with 27 pediatric patients implanted between 1987–1995 [17], 29% did not maintain full-time use in the long term. Kleijbergen [18] found a 12% rate of pediatric non-use after sequential bilateral cochlear implantation in children. Rose [19] reported non-use rates of 40%. This is not always the case, however. Raine [20] reported a non-use rate of 5% in a group of 180. Archibold [21] reported a rate of 3% among 138 pediatric users. In more recent studies, the elevated rate of pediatric non-use seems to be reduced. Markey [22] reported 2.5% of device non-users in an adolescent population, and Özdemir [6] reported 0.96% during their 11 years of experience. In our cohort we have not observed a single case of implant non-use among patients under 18 years (n = 111).

This apparent reduction in the rates of non-use among children may be related to improvements in patient selection, changes in criteria [20], being more restrictive in the indications for implantation, technological improvements in the devices themselves, and greater experience of the implantation team, all leading to improved outcomes and therefore higher motivation for continued device usage.

**Table 4:** Studies of CI non-use.

First Author, Year	Duration of the Study	Population	% Non-Users	Reasons for Non-Use
West, 1995 [23]	Unknown	Unknown	3% recipients	
Summerfield, 1995 [24]	Unknown	Unknown	3.5% recipients	Elective non-use
Archbold, 1998 [25]	3 years of follow-up	37 children	0%	
Proops, 1999 [26]	1990–1996	100 adult patients	4.0% recipients	Death unrelated to implantation, severe depression, no stimulation, iatrogenic cholesteatoma
Summerfield, 2000 [27]	1990–1998	313 adult patients	6.3% between 4–7 years after implantation 11.0% at 7.5 years after surgery	Elective non-use (medical/surgical complications, age, deaf for longer prior to implantation, low performance, low benefit, among the first 10 cases implanted by an implant program)
Spencer, 2004 [17]	1987–1995	27 prelingually children	29% recipients	Family environment, device failure, did not feel they were gaining much hearing benefit
Bhatt, 2005 [28]	1998–2002	214 adult patients	4.7% recipients	Explantation after surgical complication, comorbid illness, elective nonuse, audiologic complication, device failure
Raine, 2005 [20]	Unknown	180 children	5% recipients	Unknown
Ray, 2006 [10]	1990–2000	423 patients: 172 children 251 adults	1.89% recipients 1.18% children 0.71% adults	Children: peer pressure Adults: Depression, tinnitus, concomitant neurological problems and nonauditory stimulation
Raine, 2008 [29]	1990–2005	340 patients: 155 children 185 adults	3.8% recipients 3.2% children 0.6% adults	Children: age at implant, educational placement, and family support Adults: psychological issues and inability to adapt to the signal
Archbold, 2009 [21]	Unknown (7 years period)	138 children	3% recipients	Complex family issues, learning difficulties, experiencing pain on stimulation.
Özdemir, 2013 [6]	2000–2011	413 pediatric patients (<16 years)	0.96% recipients	Ossified cochlea due to meningitis, autism, learning disability and lack of family interest
Markey, 2015 [22]	1996–2011	79 adolescents	2.5% recipients	Complain of finding the device too loud and also suffered from headaches when wearing it, autism spectrum disorder
Kleijbergen, 2022 [18]	2014–2016	85 children receiving a contralateral CI at the age of 5 to 18 years.	12% (12 months follow-up)	The second device did not add additional benefit, lack of motivation, could not acclimatize to second implant, complaint of pain when wearing the second CI
Present study	1992–2015	316 patients: 100 children 216 adults	2.8% recipients 0% children 2.8% adults	Little or no response with the CI, refused reimplantation, cognitive problems, non-attendance of speech therapist sessions, comorbid illness, loss of audio processor

#### 4.1.1. Reason for Non-Use

Among those who became non-users in the present cohort, the reasons for non-use were diverse: two of the nine CI non-users discontinued their usage due to cognitive or linguistic issues. One user ceased using their device because they found the rehabilitation and fitting session to be incompatible with a major life change (treatment for lung cancer). One user became a non-user because of the loss of the audio processor. One user experienced a complication related to the implant function, and one derived little benefit from their device. For three users, we were unable to ascertain the reasons for their discontinuation.

It would have been ideal to attempt to draw statistical correlations between the rate of non-use and demographic, etiological, and implant-related factors. This could provide information that may help to predict which users are more likely to become non-users. Such information could be useful in candidate selection, and in the allocation of monitoring and counselling resources towards users who are more likely to become non-users. However, given the low absolute number of non-users identified during our thirty-year study period, it was not possible to perform reliable statistical analysis. As such, our analysis can only be descriptive.

The nine CI non-users had a mean age of 47.2 ± 14.3 years (range 26–70 years). This is slightly younger than the mean age of the cohort as a whole at 52.3 ± 15.6 years (range 18–88 years), but this small difference is unlikely to be meaningful. Ozdemir and colleagues [6] observed an inverse correlation in limited users (those who do not employ their device fully) between implantation age and the auditory performance improvement.

No obvious link between etiology and CI non-use was observed. Two non-users had previous episodes of meningitis. It has previously been reported that those with meningitis-associated hearing loss more frequently reject implant use, especially when other neurological sequelae are present [6,10]. However, despite this fact, in our cohort 91% of adult patients with a meningitis episode were daily CI users.

Another two non-users had suffered from bilateral sudden hearing loss; one patient was implanted one year after deafness, the other after two years of deafness. We speculate that short duration of hearing deprivation prior to implantation may have contributed to these patient’s rejection of the device; it may have been too challenging to become used to their new “ear”. In addition, one of these bilateral users suffered from mental health issues, which may also have contributed to their non-use.

It does not appear that the type of CI is related to the non-use. The non-users had a wide range of implants, from one of the oldest in the market (Nucleus 22M) to the newest available in 2015 (Concerto) (Table 2).

Several publications have investigated other causative factors contributing to non-use of CIs in children and adolescents [5,21]. These factors include peer pressure, family circumstances, behavioral difficulties, and non-support of CI use in the school environment. As no pediatric non-users were identified in our cohort, we are unable to provide evidence for or against these reports.

#### 4.1.2. Non-Use in Expanded Indications

The inclusion criteria for CI implantation have become less restrictive in recent years, being expanded to pediatric populations, those with single side deafness (SSD) or asymmetric hearing loss (AHL), and prelingually-deafened late-implanted patients. Several studies have examined the rates of non-use in these expanded indications.

Kleijbergen et al. [18] found a 12% rate of device non-users in pediatric sequentially-implanted bilateral CI users (5–18 years old) where there was a considerable delay between the first and second implantations (median 12 years; follow-up period, one year). The reasons given for the non-use included that the second device did not add additional hearing benefit, that the user lacked motivation, that the user could not become accustomed to using both implants at the same time, and that pain was present when using the second CI.

The prevalence of CI non-use in SSD has been also studied. Távora-Vieira et al. [30] observed a rate of 4.4% in a cohort who had a mean CI experience of almost one year. A higher rate of non-use (9.8%) was reported by Speck et al. [31] among adult SSD users with 6–11 years of CI experience. In children and adolescents with SSD, non-use ranged from 5% to 18% after a follow-up of greater of 12 months [32,33,34]. The main reasons for non-use were that no speech comprehension was obtained with the CI, and that there was a perceived lack of quality and persistence in rehabilitation. A recent meta-analysis reported that the duration of deafness in SSD is a significant factor, with longer durations observed among non-users [35]. In AHL, Speck et al. [31] did not observe any cases of device non-use among the 32 AHL cases interviewed at six to 11 years of follow-up.

In a systematic review of patients who are prelingually-deafened and later-implanted users, Pattisapu et al. [36] observed device non-use rates ranging from 0% to 9.5%. Bosco et al. [11] reported that prelingually-deafened adolescents are more likely to under-use their CI compared to young adults. Hearing outcomes are a critical factor determining non-use in prelingually-deafened and later-implanted users. Lammers et al. [37] observed that in a cohort of 48 users, all users who derived no benefit from their implants in daily communication eventually became non-users.

According to the former studies, it is worth noting that the rates of device non-use tend to be higher in these expanded indications than in traditional indications. This may be reflective of poorer hearing outcomes or quality of life gains in these cohorts. This should be taken into account during candidate selection and during counselling and rehabilitation.

### 4.2. Middle Ear Implant (VSB) Device Non-Users

There are few published studies that report non-use rates for VSB. Most studies report only the medical and technical results, highlighting the implant survival and complications. Table 5 documents all studies in which non-use rates for the VSB have been reported. These rates ranged from 0–13.3%. The largest cohort study thus far, with 113 individuals and 131 devices implanted, reported a non-usage rate of 2.7% [38]. This contrasts markedly with the 27.3% (6/22) non-usage rate in the present study. Several differences are apparent between that study and ours. Firstly, the mean follow-up time in Maier et al. [38] was shorter (3.4 years) than in the present study (11 years). A longer follow-up time will provide more opportunities for a user to transition to a non-user due to the users becoming fatigued or encountering life circumstances that make compliance with rehabilitation more difficult, or due to technical or medical factors such as delayed complications or device failures which may manifest only after long periods of usage. Secondly, the three non-users in Maier et al. developed hearing loss after implantation, one of whom was then explanted. In all six of our cases, non-use was due to device failure or surgical complications. Two of our non-users had conductor link extrusions (see also a previous paper of our team [39], in which it was reported that extrusion of the wire link was the main surgical complication in four out of 12 patients). This complication has previously been described in patients undergoing VSB implantation [40]. In other reports, revision surgeries were performed and hearing was restored [41]. Still others have reported that despite wire extrusion in the external auditory canal, the patients are still actively using their VSB [42].

Despite the relatively high rate of non-use among our VSB recipients, a previous study from our group has demonstrated that VSB users have high levels of satisfaction with the device [39].

Moreover, it is important to mention that five out of six of our non-users of VSB had cholesteatoma. So, as Sprinzl et al. [43] stated in their study, this special cohort needs extra counselling and caution on possible complications.

**Table 5:** Studies of VSB non-users.

First Author, Year	Years of the Study	Population	% Non-Users	Reasons for Non-Use	Mean Follow-Up	Comments
Schmuziger, 2006 [44]	2000–2002	20 patients	0%	---	42 months (range 26 to 55 months)	Two patients refused to participate in the study, and another could not be reached
Mosnier, 2008 [45]	1997–2000	100 patients	8%	Progressive hearing loss, decrease in the functional gain (revision surgery refused), poor benefit (outside selection criteria), results not correlated to expectation, psychological problems, refusal to pay for processor repair, device failure in evaluation	Six years (range, 5–8 years)	In addition to what the authors consider to be non-users, three subjects were deceased, three were lost to follow-up, and seven were explanted without reimplantation.
Zwartenkot, 2013 [46]	1997–2010	39 patients	13%	Insufficient benefit and device problems	7.5 years, minimum two years	Also included Otologics MET middle ear implant systems
Maier, 2015 [38]	1997–2012	113 patients (and 131 VSB implantations)	2.7%	No hearing benefit from the implant	3.4 years (range 0–13.9 years)	---
Jones, 2021 [47]	2011–2017	15 patients	13.3%	No device beneficial	13 months, with a minimum of three months	This study also included BB implants
Present study	2008–2015	22 patients: 0 children 22 adults	22.7%	Device failure or surgical complication	11 years (range 7–15 years)	

### 4.3. Bone Conduction Implant (BB) Device Non-Users

Among our cohort of BB users (n = 18), we did not observe any cases of discontinuation during our follow-up period (2012–2022). However, it was observed that patients with SSD (n = 2) have attended fitting sessions less frequently during the last two years. After contacting them, they told us that their use of their BB device had decreased, due to less social contact during the COVID-19 pandemic. As they used the device for at least 2 h per day, we did not consider them to be device non-users [48].

There are not many studies regarding BB device use. In 2019, Brkic et al. [49] stated that to that point, there had been no studies about BB usage rates with a long follow-up. They reported that after a mean follow-up of 2.3 years, 10.9% of the implantees (seven out of 64) were not using the device. The reasons were explantation because of skin dehiscence (n = 3), wound dehiscence (n = 1), lack of benefit from implantation due to the indication criteria (n = 1), and reimplantation with a CI due to progressive hearing loss (n = 2).

Han et al. [48] reported the non-use rates of their patients with bone conduction devices. Among those implanted between 2013–2017, about 40% of those in the SSD group used the device less than 2 h per day. For six of the non-users, the reason was a self-reported limited benefit of the device, and for one non-user, the reason was that the audio processor was lost. Moreover, 10% of users with mixed hearing loss were device non-users due to wound infection. Garcier et al. [50] and Jones et al. [47] both reported that all patients were users at the last follow-up; the mean follow-up times were nine and 13 months, respectively.

Due to the fact that we have focused our study on transcutaneous hearing devices, patients with BAHA systems have not been included. Anecdotally, we have observed a non-usage rate of 40.9% among BAHA users with radical cavities. Infection, fixture losses, and poor sound quality were some of the reasons for non-use. This apparent high rate of non-use among BAHA recipients warrants further analysis.

## 5. Conclusions

In our cohort, the rates of auditory implant non-use vary among the different implant types: 0% in bone conduction implant (BB) users, 2.8% in CI patients, and 27.3% in middle ear implant (VSB) users. The reasons for non-use are multifaceted. Because of the small proportion of non-users, information about what the predictive factors are could not be provided. Continued follow-up and contact with patients is essential to facilitate long-term continued use of their devices.

## Figures and Tables

**Table 1:** Demographic details of the subjects included in the study. CI: cochlear implant; MEI: middle ear implant; VSB: Vibrant SoundBridge; BCI: bone conduction implant; BB: BoneBridge.

	n (%)	Age at Implantation (Years) (Mean ± SD, Range)	Period of Surgeries	Time since Surgery (Years) (Mean ± SD, Range)
***CI***	316		1992–2015	15.1 ± 5.4 (7.1–30.6)
Aged < 18 years	100 (32%)	3.8 ± 3.8 (0–16)		14.2 ± 5.0 (7.1–30.0)
Aged ≥ 18 years	216 (68%)	52.3 ± 15.6 (18–88)		15.5 ± 5.5 (7.3–30.6)
***MEI (VSB)***	22		2008–2015	11.4 ± 2.1 (7.2–14.9)
Aged < 18 years	0 (0%)			
Aged ≥ 18 years	22 (100%)	51.2 ± 14.2 (27–77)		11.4 ± 2.1 (7.2–14.9)
***BCI (BB)***	18		2012–2015	8.6 ± 1.1 (7.1–10.5)
Aged < 18 years	1 (6%)	17.0 ± 0.0		8.3
Aged ≥ 18 years	17 (94%)	50.9 ± 16.5 (18–71)		8.6 ± 1.2 (7.1–10.5)

**Table 2:** Clinical details of Cochlear Implant non-users. F, female; M, male.

Patient	Gender	Year of Surgery	Age at Implantation	Time of Hearing Loss	Etiology	Duration of Hearing Loss	Electrode Insertion	Type of CI	Comments
1	F	1994	46	Postlingual	Sudden hearing loss	2	Incomplete	Nucleus 22M	Not attending fitting sessions because the patient does not observe any benefit. No good hearing outcomes were reached, and the patient decided not to use the audio processor
2	M	2001	59	Postlingual	Meningitis	54	Complete	Combi40+	Claims it is a waste of time to use the CI. A documented reason was not found in her medical records
3	M	2001	38	Postlingual	Meningitis	0	Complete	Combi40+	No response with the CI, probably because the auditive nerve is affected
4	F	2003	53	Postlingual	Unknown	17	Complete	Combi40+	Audio processor was lost 19 y after implantation, and patient had no money to buy a new one
5	F	2005	60	Postlingual	Meningitis	48	Complete	Pulsar	A patient’s relative was not able to give a definite reason for device non-use after contacting her by phone
6	M	2005	39	Postlingual	Otosclerosis	14	Complete	Pulsar	Mechanical stress on the implant. The patient refused reimplantantion surgery 12 years after surgery
7	M	2007	34	Postlingual	Sudden hearing loss	1	Complete	Pulsar	The patient suffered from mental problems several years after the implantation
8	F	2008	26	Postlingual	Unknown	13	Complete	Pulsar	Problems with the language (Chinese patient) when attending the rehabilitation sessions; never became used to the CI
9	M	2013	70	Postlingual	Unknown	52	Complete	Sonata	Treatment for lung cancer just after the first fitting session. He refused to use the CI and focused on cancer treatment

**Table 3:** Clinical details of Vibrant Soundbridge device non-users. F, female; M, male.

Patient	Gender	Year of Surgery	Age at Implantation	Type of Hearing Loss	Etiology	Comments
1	F	2008	38	Conductive	Cholesteatoma	Explantation in another country (France). Unknown problem.
2	F	2009	64	Mixed	Cholesteatoma	Cable extrusion. Currently using a cochlear implant.
3	M	2010	31	Mixed	Cholesteatoma/Chronic otitis media	Problems with the coupling one year after the implantation. They were reimplanted, but no improvement occurred.
4	F	2011	77	Mixed	Cholesteatoma	Cable extrusion five months after surgery. The patient refused a new implant.
5	M	2014	71	Mixed	Chronic otitis media	No response in the first fitting session. Currently wearing a cochlear implant.
6	F	2010	40	Conductive	Cholesteatoma	Reimplantation with a BB implant due to recurrent cholesteatoma

## Data Availability

The data presented in this study are available on request from the corresponding author.
